# The Methods of Microscopical Examinations and of Artificial Culture of Bacteria

**Published:** 1886-04

**Authors:** George C. Hummel

**Affiliations:** Savannah, Ga.


### THE METHODS OF MICROSCOPICAL EXAMINATION AND OF ARTIFICIAL CULTURE OF BACTERIA.

BY GEORGE C. HUMMEL, M. D., SAVANNAH, GA.

READ BEFORE THE THIRTY-SIXTH ANNUAL SESSION OF THE MEDICAL ASSOCIATION OF GEORGIA AT SAVANNAH, APRIL 15, 1885.

### NUMBER I.

The attentive observer finds in our daily surroundings, which are the object of hygienic research, widely propagated organisms almost invisible even to the eye, armed with the most perfect optical instruments. They play nevertheless a most important part in the household of nature and the existence of man. In every branch of hygiene we feel their influence. In the air, the ground and the water we find these smallest organisms, which we are able to recognize in our nearest surroundings, our homes and our foods as continual companions and often as dangerous enemies.

They have been variously named by different authors. From a physiological and chemical point of view, the designation as organized ferments would be proper. Pathologists have named them vegetable parasites. Let us speak of them with the most comprehensive term, as micro-organisms or microbes.

I shall not encroach upon your valuable time by giving you an exhaustive description of their morphology and biology, of which you may derive all the knowledge you desire from a very large literature. My intention is to make you acquainted by actual demonstration—the only way which promises success—with the methods of microscopical examination and artificial culture of those micro-organisms, which as producers of disease interest the pathologist. These are the hyphomycetes or moulds, the blastomycetes or yeasts and the schizomycetes or bacteria.

The moulds play an unimportant part as producers of disease man. The high temperature, the richness in albumen and the only slightly alkaline juices of the body are unfavorable to them, and the very large amount of oxygen which they need for their fructification is not sufficiently supplied in the internal organs. They seldom occur in the human body, and if they do can only be considered as secondary growths, developing on the dead tissue, produced by antecedent morbid processes and not as the specific causes of the diseases in question.

Of the yeasts also only a few claim our attention, like the sacche-ramyces allircan, which is thought to be the cause of thrush; yet this theory is not an established one, as in this frequent disease of infancy a great many other fungi are found in the mucous membranes of the mouth and pharynx.

The large intestines often contain a vast number of yeast cells, and if the food taken contains much saccharine matter fermentation may be kept up for a long time, a condition which will certainly engage the attention of the physician.

Of the greatest importance are, in contrast to the two classes named, the schizomycetes, or, as they are commonly called, bacteria, and it is to this class I call your attention. To study them thoroughly the first condition is to master the methods of microscopical examination and their artificial culture.

It was my good fortune to study bacterioscopy, under the guidance of Prof. Feugge, of Gœttingen, a high authority in this comparatively new branch of pathology, and a warm adherant and friend of the celebrated Dr. Koch, of Berlin, with whose recent investigations of bacillus of tuberculosis and of cholera you all are acquainted.

Prof. Feugge's works have been extensively used in compiling this paper. Let us at first consider the microscopical examination.

Almost everything around us may be subjected to microscopical examination: Fluids and solid materials, articles of food, samples of earth, dust, or vegetable and animal organs and juices taken from the living or the dead.

If we want to examine a specimen for bacteria, it must be our first endeavor to prevent microbes, growing in material surrounding them, from entering into the specimen. To do this we must destroy any micro-organisms clinging to all instruments, glasses or fluids that are needed in preparation of specimens, by exposing the first two named, for a short while, to a dry heat of at least 150° C.=302° F., or in many cases by immersion into an aqueous solution of corrosive sublimate in the proportion of 1 : 2000. The fluids should be exposed for some time to steam at 100° C.=212° F. If we look for pathogenic bacteria, we must be aware of the fact, that on the surface of every living being, as on the skin, in the mouth, etc., myriads may be found. After death these spread rapidly into the tissues, but specimens containing pathogenic bacteria should never be taken from an unclean surface, even of the living subject. After death the interior of organs ought to be opened, if possible, only by fresh cuts with recently heated knives.

The direct microscopical examination (eventually with the addition of a ¾ per cent. solution of chloride of sodium, a mixture of glycerine and water, a 10 per cent. solution of acetate of potassium, etc., etc.) is only successful in the larger varieties. Smaller bacteria can often not be perceived even with the highest power, especially if other materials, such as cells, cell nuclei, cell detritus, crystals and amorphous inorganic substances, are present. Therefore, if a thorough investigation of the specimen is attempted, the staining of the same with suitable dyes is indispensable. Most bacteria have a peculiar affinity for dyes, so much that often the bacteria alone are stained, or at least in deeper shades than the other material. Only with the aid of the staining process are we enabled to decide with certainty that bacteria are absent in a given specimen.

The methods of staining are different in fluids and in animal tissues.

Fluids are thus prepared for examination : With a platinum wire that has been carefully brought up to a red heat and cooled again just before using, a small drop of the fluid is taken up and spread in a thin film over a coverglass. Of pus, sputa and other slimy masses, a very minute portion is pressed between two coverglasses, which are immediately drawn from each other by a gliding motion. In a few moments the specimen will be dry. To fix it on the cover-glass, pass it three or four times quickly through a flame of a gas or alcohol burner. After this place the glasses on a piece of blotting paper and apply the dye with a dropper. Let dye remain on specimen five or ten minutes, then suck it up with a bit of blotting paper and wash the specimen several times in distilled water, or in very dilute acetic acid (about one drop of the acid to two ounces of water). The specimen may now be examined at once in a drop of water, or it is dried and mounted in Canada balsam in the usual manner.

To search for bacteria in tissues and organs, it is necessary to make very thin sections, containing only one layer of cells, if possible. This must be done with a microtome. The selection of an instrument which will last is a difficult matter. The one I now use is manufactured by Katch, of Munich, and is by far the best one I have ever used. It cuts both fresh tissues, frozen with the ether spray, or specimens hardened in alcohol or any other hardening fluid. Fresh sections are, immediately after they have been cut, put in a ¾ per cent. solution of chloride of sodium, thence in absolute alcohol, the hardened specimens at once in the alcohol. From the alcohol they are placed in the dye, in which they have to remain usually from half an hour to five hours, in some instances as long as 24 hours. If the cuts are taken out of the dye, we find the whole tissue deeply stained. For the purpose of differentiation the dye has to be taken out of the tissue as much as possible, leaving only the bacteria deeply stained. This is done by washing the sections for several minutes in alcohol. They are then made transparent in oil of cloves. If while in the oil much coloring is yet given off, they are again put in fresh absolute alcohol and back in the oil. They may now be directly examined in the oil of cloves, or if intended to be preserved, the oil is carefully sucked up by blotting paper, and the specimens are mounted in Canada balsam.

Some dyes do not keep if treated with oil of cloves, and if the specimens are intended for preservation, oil of bergamot is a good substitute.

The basic or neutral aniline dyes are the most suitable, as they have also a great affinity for the cell nuclei. A few other colors, as hæmatoxylon, carmin, picrocarmin, etc., are also used. Bacteria are dyed red with carmin, purpurin, fuchsin, magdala and magenta; brown with bismarkbrown, or vesuvin; green with methylgreen, iodine green; blue or violet, with hæmatoxylon, methylenblue, iodine, violet, methyl, gentian violet and dahlia.

The most universally used dye for fluids, as blood, pus, discharges from the genital organs, liquids from cutaneous eruptions, etc., is the methylenblue thus prepared.

Add thirty parts of a saturated alcoholic solution of methylenblue to 100 parts of an aqueous solution of caustic potash in the preparation of 1:10,000. Filter before using.

Some bacteria show an affinity for only a few dyes; therefore, to find yet unknown microbes, the different dyes have to be experimented with.

For the purpose of differentiation between cell nuclei and bacteria double staining is advisable. Picrocarmin and gentian violet suit well for the purpose, because the picrocarmin will take the violet tint out of the nuclei, while the bacteria keep a beautiful violet color. The cuts are put into an aqueous solution of gentian violet (1:200) at the temperature of 38° C.=100° F., for about 4 hours, washed successively in water, alcohol, water; then they are placed into a solution of picrocarmin for several hours; again washed in water and alcohol, put in oil of cloves, alcohol, oil of cloves (or bergamot), and mounted as before. This is a beautiful process for specimens containing bacteria of chicken cholera and of the septicæmia of rabbits, or the fine bacilli of septicæmia. The microbes appear violet and the tissues red.

The solution of picrocarmin is prepared in the following manner:

Dissolve 2 parts of best carmine in 200 parts of aqua ammonia and set aside for 24 hours; add 200 parts of saturated watery solution of picric acid and set aside for 3 days. Always filter before using. If too yellow add a few drops of dilute acetic acid; if too red of aqua ammonia.

It will be perhaps of use to describe the staining of the bacilli of tuberculosis and of the micrococci of pneumonia, as they have a process quite peculiar to themselves. Specimens of sputa suspected of containing the former are thus prepared:

Shake 50 parts of water with 5 parts of aniline oil thoroughly and filter. Into the filtered solution drop a saturated alcoholic solution of fuchsin or gentiana-violet until the fluid becomes slightly turbid (about 10 to 20 drops of the dye to 3 drachms of aniline water). Fill watch-glass with the dye and heat over alcohol lamp until some steam is given off. Float the cover-glass, upon which the sputum has been spread, for a few minutes on the dye. Wash in alcohol, dilute nitric acid (1:3), alcohol until the dye is apparently washed out, rinse off in distilled water and float for a short while (about half a minute) on a watery solution (½ per cent.) of methylenblue if fuchsin has been used or bismarkbrown if gentiana has been used. Wash in water, dry and mount. Cuts have to be left in the fuchsin or gentiana solution for 24 hours, washed in 60 per cent. alcohol, dilute nitric acid (1:3), 60 per cent. alcohol, floated for a few minutes on a solution of methylenblue or bismarkbrown, washed again in 60 per cent. alcohol, immersed in absolute alcohol, cleared in oil of cedar and mounted. The bacillus of the pearly disease of cattle, which is identical with that of tuberculosis, is dyed in the same way; also that of leprosy.

Sections containing micrococci of pneumonia are prepared as follows: Place in a watery solution of gentiana (1:200) for five minutes, then also for five minutes in the officinal Lugol's solution, diluted with distilled water, until the color is that of sherry, quickly steep in absolute alcohol, keep in oil of cloves for half an hour and mount.

For the examination of specimens containing bacteria, good high powers are essential; at the least a power magnifying 300-400 linear diameters, 1-12 or 1-14 oil immersion will be found sufficient. A sub-stage condenser, such as Abbé's, is absolutely necessary.

The best pictures of specimens are obtained by photography. The brown stains are preferable for that purpose.

